# Complete Genome Sequences of Four Pseudomonas aeruginosa Bacteriophages: Kara-mokiny 8, Kara-mokiny 13, Kara-mokiny 16, and Boorn-mokiny 1

**DOI:** 10.1128/mra.00960-22

**Published:** 2022-11-14

**Authors:** Andrew Vaitekenas, Anna S. Tai, Joshua P. Ramsay, Stephen M. Stick, Patricia Agudelo-Romero, Anthony Kicic, WAERP, AREST CF

**Affiliations:** a Occupation, Environment and Safety, School of Population Health, Curtin University, Perth, Western Australia, Australia; b Wal-Yan Respiratory Research Centre, Telethon Kids Institute, The University of Western Australia, Perth, Western Australia, Australia; c Department of Respiratory and Sleep Medicine, Sir Charles Gairdner Hospital, Nedlands, Western Australia, Australia; d Institute for Respiratory Health, Nedlands, Western Australia, Australia; e Faculty of Health and Medical Sciences, The University of Western Australia, Perth, Western Australia, Australia; f Curtin Health Innovation Research Institute and Curtin Medical School, Curtin University, Perth, Western Australia, Australia; g Department of Respiratory and Sleep Medicine, Perth Children’s Hospital, Perth, Western Australia, Australia; h Centre for Cell Therapy and Regenerative Medicine, School of Medicine and Pharmacology, The University of Western Australia and Harry Perkins Institute of Medical Research, Perth, Western Australia, Australia; i Australian Research Council Centre of Excellence in Plant Energy Biology, School of Molecular Sciences, The University of Western Australia, Perth, Western Australia, Australia; j European Virus Bioinformatics Center, Jena, Thuringia, Germany; Queens College CUNY

## Abstract

Pseudomonas aeruginosa is an opportunistic pathogen. Here, we report the isolation of four bacteriophages from wastewater. All four bacteriophages belong to the *Myoviridae* family. Kara-mokiny 8, 13, and 16 are of the *Pbunavirus* genus and have genomes between 65,527 and 66,420-bp. Boorn-mokiny 1 is of the *Phikzvirus* genus and has a 278,796-bp genome.

## ANNOUNCEMENT

Pseudomonas aeruginosa is an opportunistic pathogen that frequently causes burn wound (1), nosocomial (2, 3), and airway infections (4–6). Treatment is complicated by its high intrinsic antibiotic resistance, biofilm formation, and ability to acquire resistance (7). Classified as a critical priority pathogen (8), P. aeruginosa is now a primary target for bacteriophage (phage) therapy. Here, we report complete genomic sequences of four lytic phages isolated from wastewater specific for P. aeruginosa.

Phages Kara-mokiny 8, Kara-mokiny 13, Kara-mokiny 16, and Boorn-mokiny 1 were isolated from a suburban wastewater treatment plant in Perth, Australia, using P. aeruginosa isolates M1C37, M1C74, M1C108 (derived from children with cystic fibrosis [CF]; AREST CF, Melbourne, Australia) and the PAO1 reference strain, respectively. Phages were purified by three rounds of single-plaque isolation and propagation on agar plates using their respective hosts (9). Phage genomes were extracted by DNase I and RNase A treatment before proteinase K capsid destruction and purification using the Qiagen (Hilden, Germany) DNeasy blood and tissue kit (10). DNA libraries were prepared using a Nextera XT kit (Illumina, San Diego, CA, USA) and sequenced on the Illumina NovaSeq 6000 (Illumina) platform by the Australian Genomic Research Facility (Victoria, Australia), generating 150-bp paired-end reads. We used an ultradeep sequencing approach (coverage, >1,000×) which was complemented by a robust annotation and analysis pipeline (11). Software, versions and parameters used have been provided in a publicly available Docker image (https://github.com/JoshuaIszatt/phanatic; v1.0.3). Briefly, adapters and poor-quality sequences were removed by Trimmomatic (HEADCROP: 15) (12). Kara-mokiny 8, 13 and Boorn-mokiny 1 were then deduplicated (ac=f s = 10 e = 7 minidentity = 99) and normalized (min = 5 target = 500) and reads were merged (-ea mininsert = 125 minoverlap = 20) (13), followed by *de novo* assembly using SPAdes (-t 12 -m 5 –only-assembler –careful -k 21,33,55,77,99,127) (14). Kara-mokiny 16 reads surviving adapter trimming were assembled by Unicycler v0.4.8 in bold mode (15) and used as an untrusted contig for merged-read assembly using SPAdes v3.15.4 (14). Assembly quality and completeness were evaluated using Quast (16) and CheckV (17), respectively. Genomes were annotated using Prokka with the Prokaryotic Virus Remote Homologous Groups (PHROGS) database, and bacterial genes were searched for using Abricate (18). Nucleotide similarity searches using NCBI databases were performed with BLASTn (19). JSpecies (20) was also used to determine the average nucleotide identity (ANI) percentage of each phage to their closest relative. Default parameters were used in all software used unless specified.

The ANIs of Kara-mokiny 8, Kara-mokiny 13, and Kara-mokiny 16 indicate that they belong to the *Pbunavirus* genus, while Boorn-mokiny 1 belongs to the *Phikzvirus* genus (Table 1). Interestingly, Kara-mokiny 13 had an ANI of <95% (Table 1), indicating that it is a new phage species of *Pbunavirus*. The reported phages’ genomic and physical characteristics make them therapeutic candidates requiring further investigation.

**TABLE 1:** Characteristics of isolated phages

Parameter	Data for indicated phage			
	Kara-mokiny 8	Kara-mokiny 13	Kara-mokiny 16	Boorn-mokiny 1
Genome size (bp)	66,420	65,527	66,600	278,796
No. of reads used in assembly	187,247	183,933	250,250	794,559
Postnormalization genome coverage (×)	588	587	754	585
CheckV completeness (%)	100	99.06	100	100
CheckV quality	High	High	High	High
GC content (%)	55.68	55.20	54.76	36.91
No. of CDS^*a*^	91	92	93	355
No. of hypothetical proteins	55	56	55	268
No. of tRNAs	0	0	0	6
No. of antibiotic resistance genes	0	0	0	0
No. of bacterial virulence genes	0	0	0	0
Closest related phage (GenBank accession no.)	Pseudomonas phage PASA16 (MT933737.1)	Pseudomonas phage PAP8 (OL754588.1)	Pseudomonas phage PA4 (MZ285878.1)	Pseudomonas phage vB_PA32_GUMS (MN563784.1)
ANI (%)	97.26	94.29	97.18	98.82
GenBank accession no.	OP310974	OP310979	OP310982	OP310983
Sequence read archive accession no.	SRX17294654	SRX17295164	SRX17298365	SRX17298366
BioProject accession no.	PRJNA862681	PRJNA862681	PRJNA862681	PRJNA862681

a CDS, coding DNA sequence.

### Data availability.

Complete phage genomes were deposited in the GenBank and NCBI Sequence Read Archive databases under the accession numbers in Table 1.
